# Supplementary material for: Are carbon emissions trading and green financial instruments synergistic? -Comprehensive quantitative research based on content analysis
Source: PLoS One. 2024 Mar 7;19(3):e0298601. doi: 10.1371/journal.pone.0298601 (PMC10919601; doi:10.1371/journal.pone.0298601)
Supplement: S1 Data — (ZIP) [file pone.0298601.s001.zip › Data/地级市Z_F工作报告.zip!/║╙─╧/┬σ╤⌠╩╨/└┤╫╘╣1⁄2╓┌║┼í╛┬φ┐╦╩2╛▌═°í┐_2014.docx]

各位代表：

现在，我代表市人民政府向大会作工作报告，请予审议，并请各位政协委员和列席人士提出意见。

一、过去五年工作回顾

本届政府任期的五年，是洛阳发展史上极不平凡的五年。在省委、省政府和市委的正确领导下，在市人大、市政协的监督支持下，市政府与全市人民一道，坚持以邓小平理论、“三个代表”重要思想、科学发展观为指导，深入学习贯彻党的十七大、十八大精神，克难攻坚，扎实工作，圆满完成了市第十三届人大一次会议确定的各项目标任务。

这五年，我们克难求进稳增长，综合经济实力实现新跨越。面对全球金融危机带来的严峻形势，我们认真贯彻落实中央、省各项决策部署，坚持项目引资双带动，大力实施“六加一”攻坚战，突出抓好“开放招商、服务企业、优化环境”等重点工作，先后制定了应对危机稳增长的一揽子政策措施和行动计划，在极为困难的情况下实现了经济平稳较快增长。五年间，全市地方公共财政预算收入、固定资产投资、社会消费品零售总额等主要经济指标实现翻番。地方公共财政预算收入由116.6亿元增加到234亿元；固定资产投资由974.3亿元增加到2589亿元；社会消费品零售总额由577.1亿元增加到1257.7亿元。2013年，全市生产总值达到3140.8亿元，年均增长11.2%。

这五年，我们加快转型调结构，产业升级取得重大突破。坚持项目带动、创新驱动，加快产业集聚区等载体建设，大力推进老工业基地振兴转型。改造提升传统优势产业。制定重点产业调整振兴规划和产业链图谱，以十大园区、四大企业搬迁改造为重点，通过战略重组、技术改造等方式，推动传统产业升级。五年共实施亿元以上重大产业结构调整项目1973个，中信重工新重机、万基控股40万吨铝板带箔一期等项目建成投产，新增铝精深加工产能110万吨，建成了国内唯一完整的钛产业链条，一批超临界的火电机组取代了小火电，光伏电站、风电场项目实现了零的突破。培育壮大战略新兴产业。围绕智能装备制造、电子信息等重点领域编制产业规划，累计实施重点项目389个，景安IDC云计算产业园、中航光电技术产业基地、普莱柯动物疫苗产业化等一批项目加快实施或竣工投产，我市入选国家硅材料及光伏高新技术产业化基地、新材料产业国家高技术产业基地。提速发展现代服务业。五年累计实施重大服务业项目469个，会展中心、建业·凯旋广场等一批大型会展、商业综合体建成开业，宏进农产品物流园区等一批物流项目建成投运，16个商务中心区、特色商业区加快建设。隋唐城明堂、天堂等大遗址保护展示工程建成开放，牡丹文化节升格为国家级节会，我市被评为“中国牡丹花都”。新增5A级旅游景区4家，旅游人数、旅游总收入年均分别增长13.6%和17.4%，我市成为全国旅游标准化试点城市。中信、兴业、民生等25家金融机构落户我市，村镇银行基本实现县域全覆盖，资本市场融资水平进一步提高。房地产市场健康发展，完成投资、商品房销售面积年均分别增长22.3%和21%。持续增强科技创新能力。加快产学研军融合发展，五年建立市级以上研发平台275个，实施国家、省科技项目492项，荣获省以上科技奖177项，申请专利2.4万件。新当选院士1名，引进“千人计划”1人，培养中原学者6名。中信重工、一拖集团等企业荣获省长质量奖，725所荣获首届中国质量奖提名奖。高新技术产业增加值占生产总值的比重由4.9%提高到9.8%。我市荣膺首批国家创新型试点城市、首批国家知识产权示范市、国家军民结合新型工业化产业示范基地，连续四次被评为全国科技进步先进市。大力推进节能减排。严格项目准入，淘汰落后产能，五年单位生产总值能耗累计下降21.3%。积极创建国家环保模范城市，强力实施碧水蓝天工程，火电机组全部建成脱硫设施，涧西、瀍东污水处理厂得到改造提升，新区污水处理厂建成投运，在全省率先完成县县建成污水处理厂的任务，主要污染物总量减排目标全面完成。我市荣膺全国节能减排二十佳城市、可再生能源建筑应用示范城市称号。

这五年，我们拓展提速抓城建，城市功能品位显著提升。坚持高水平规划、高标准建设、高效能管理，新一轮城市总体规划、土地利用总体规划成功获批，城市建成区扩展到210平方公里，形成了先跨洛河、再跨伊河发展的格局，全市城镇化率达到49.4%。进一步拉大新区框架。新区规划面积拓展至515平方公里，五年完成投资1141亿元，初步形成了核心区、伊滨新区两个城市组团。核心区市政路网及配套设施基本实现全覆盖，龙门大道改造、洛宜铁路改线、博物馆、高铁龙门站等一批标志性工程竣工投用。伊滨新区主干路网框架已经形成，伊河治理初见成效，水、电、通讯、热力等配套设施投入使用。加快洛北城区改造。累计开工旧城、城中村、棚户区改造面积7721万平方米、建成2835万平方米，改善了12.9万户居民的居住条件。新建、改造道北五路、滨河北路等市政道路86条，路网结构更加合理。瀛洲桥、凌波桥等4座桥梁竣工通车，市区南北通行能力明显提升。新增供热面积927万平方米。增强基础支撑能力。坚持对外建设大通道、对内完善大循环，洛阳至西安、北京、深圳等城市的高铁通车运行；连霍高速洛阳段扩宽改造、郑卢高速洛阳段、洛栾高速以及洛伊、洛偃、洛宜、洛吉一期快速通道建成通车，实现了县县通高速，半小时经济圈初步形成；新310国道、市区环城路等一批项目开工建设；洛阳机场航站楼改扩建工程竣工投用，民航客运吞吐量突破70万人次。西气东输二线入市管线、中石油安－洛线入市管道建成通气，结束了我市没有管道天然气的历史。洛阳西500千伏变电站等工程建成投用，新增110千伏以上变电站19座，各县（市）均实现了110千伏双电源供电。提高城市管理水平。坚持条块结合、重心下移，加强城市精细化管理，形成了“两级政府、三级管理、四级网络”的管理格局。数字化城市管理系统一期建成投运，实现了城区全覆盖。对498个老旧居民小区和185条背街小巷进行了综合整治。新开通公交线路22条，新购公交车辆1302台。环卫基础设施更加完善。加大生态建设力度，城市绿化覆盖率由34.4%提高到37.9%。“四河七渠”治理成效明显，水系品位不断提升。我市荣膺全国文明城市、国家卫生城市、国家森林城市称号。

这五年，我们强基固本惠“三农”，农民生产生活条件不断改善。加快农业结构调整。巩固提高粮食综合生产能力，大力发展畜牧业、蔬菜、林果、花卉苗木等高效农业。积极实施农业产业化集群培育工程，宜阳伊众肉牛、偃师众品生猪、栾川君山中药材等入选省级产业化示范集群。着力打造十大精品休闲园区和四大休闲农业精品线路，栾川、嵩县被确定为全国休闲农业示范县。积极培育各类农业经营主体，新增产业化龙头企业130家、家庭农场157家。改善农村基础条件。加快推进农田水利建设，除险加固病险水库137座，全面启动前坪水库前期工作，新增有效灌溉面积23.6万亩，解决饮水安全154万人。硬化通组通户道路11487公里，行政村客车通达率99%。实施农电素质三年提升工程，农村电网保障能力明显增强。农业机械化水平达到65%。美丽乡村示范点建设稳步推进，农村环境整治成效显著。植树造林268万亩，森林覆盖率由42%提高到47%。治理水土流失面积700平方公里，我市荣获全国首个水土保持生态文明城市称号。加大扶贫开发力度。大力实施搬迁扶贫、整村推进和连片开发，积极开展产业扶贫、社会扶贫，建设搬迁扶贫新村180个，实施整村推进350个，9.2万余名贫困群众喜迁新居，减少贫困人口29.9万。

这五年，我们深化改革促开放，发展活力进一步增强。强力推进各个领域的改革。以产权制度改革为核心，通过股权划转、增资扩股、资产转让等形式，积极开展与优势央企（省企）的战略合作。对洛轴集团等四家企业实施政策性破产，推进黄河水泥等四家企业依法破产，妥善解决了破产企业职工的养老、医保等历史遗留问题。启动新一轮国企改革和事业单位分类改革，扎实推进国家公立医院改革试点城市建设，38家企业、325家事业单位完成改革任务。稳步推进投融资体制改革，优化整合政府投资公司，整顿规范融资类担保投资公司。市本级道路运输管理体制改革基本完成。税收征管改革和营改增试点工作正式启动。排污权有偿使用试点工作有序推进。集体林权配套制度改革步伐加快。加大开放招商力度。围绕“4＋2”重点产业，建立完善大员上阵、驻地招商、差别化考核、专项述职等招商引资工作机制，五年签约亿元以上项目1193个，累计到位资金2605亿元。成功引进了惠普－洛阳国际软件人才及产业基地、中兴通讯智慧产业基地等一批龙头项目，麦达斯铝业5万吨轨道车型材及车体大部件、龙鼎铝业60万吨高精度铝板带箔、四季沐歌太阳能工业园等一批项目竣工投产。在洛投资的世界500强、国内500强企业分别达到24家和35家。对外贸易迈上新台阶，五年累计完成外贸进出口总额80亿美元，年均增长12.4%。发展壮大非公有制经济。出台了一系列鼓励非公有制经济发展的政策措施，大力培育龙头企业和民营企业家群体，非公有制经济占生产总值的比重较2008年提高7个百分点，营业收入超亿元企业由150家增加到330家。

这五年，我们改善民生促和谐，城乡居民享受到更多实惠。坚持办好省定、市定惠民实事，下大力气保障和改善民生。五年来，全市各级财政用于民生的支出达980亿元，年均增长18.1%，占地方公共预算支出的67.6%。城镇居民人均可支配收入和农民人均纯收入分别达到24820元和8756元，年均分别增长11.1%和13.8%。优先发展教育事业。实施农村寄宿制学校建设、薄弱学校改造、取暖降温、“班班通”等重点工程，新建、改建中小学208所、幼儿园761所，城市区义务教育基本消除超大班额。引进北京第二实验小学、枫叶国际学校等教育品牌，新建各类民办学校248所。实施高中阶段提升工程，普通高招上线人数连创新高，一本上线人数实现翻番。新区教育园区初具规模，洛阳师范学院等6所学校顺利入驻。职业教育快速发展，4所职业院校成功升格。加强就业再就业工作。累计发放小额担保贷款70亿元，新增城镇就业67万人，年均实现农村劳动力转移就业140万人。提升社会保障能力。全面启动城乡居民社会养老保险，参保率达到95%。企业退休人员每月人均养老金从1104元提高到1758元。医疗、失业、工伤、生育等各类保险覆盖率不断提高。城镇居民大病医疗保险和新农合大病保险试点顺利实施。逐年提高保障标准，保障城乡低保对象33.4万人。开工建设保障性住房1176万平方米，竣工821万平方米，发放租赁补贴1.6亿元，解决了11.5万户低收入家庭的住房困难。改造农村困难群众危房8.1万户。增强公共卫生、文化服务功能。实施优势医院倍增计划，新增床位5410张。城区居民15分钟就医服务圈初步形成，所有行政村全部建成标准化村卫生室，乡镇卫生院改造任务全部完成。在全省率先实现县域公立医疗机构药品零差率销售全覆盖、首家推行“先看病、后付费”诊疗服务模式。乡镇文化站、农家书屋等公共文化服务网络实现城乡全覆盖，公共文化场馆免费对外开放。文化惠民活动和全民健身活动蓬勃开展，“河洛欢歌”广场文化狂欢月活动荣获文化部群星奖。成功举办了河南省第十一届运动会、洛阳市第十二届运动会。维护社会和谐稳定。加强平安洛阳建设，连续四年被评为全省平安建设先进市。深入排查化解矛盾纠纷，畅通群众诉求渠道，信访形势平稳向好。持续抓好安全隐患排查整治和“打非治违”，安全生产形势稳定。加强食品药品市场监管，深入开展专项治理整顿，保障了群众食品药品安全。坚持打防并举，始终保持对违法犯罪活动的高压态势，社会大局和谐稳定。

这五年，我们转变作风提效能，服务型政府建设取得新成效。依法接受人大及其常委会的法律监督和工作监督，自觉接受政协的民主监督，办理人大代表议案、建议1199件，政协委员提案1867件，满意率均达98%以上。提请审议地方性法规草案12部，出台、修订政府规章37件。深入开展“环境创优年”、“作风转变年”等活动，持续清理行政审批事项，优化再造审批流程，审批时限平均压缩32%。市行政服务中心请办事项办结时限降至8天以内。建立完善110城市应急联动服务平台，联动服务单位发展至108家，群众诉求办结率和满意率保持在95%以上。深入开展“电视问政”、“群众评诺”等活动，一大批群众关注的热点问题得到有效解决。扎实开展“双拥”工作，加强民兵预备役建设，连续六次荣膺全国“双拥模范城”称号。与此同时，人口计生、民族宗教、对台、外事侨务、统计、审计、人防、气象、地震、档案、史志、残疾人工作、妇女儿童事业等都取得了新的成绩。

看似寻常最奇崛，成如容易却艰辛。各位代表！过去的五年，面对新世纪以来最严峻的挑战，我们团结拼搏，砥砺前行，走过的历程充满艰辛，取得的成绩令人振奋，积累的经验弥足珍贵。五年来，我们始终坚持发展为要，持续转型。把加快发展、科学发展作为破解一切问题的关键，不断做大总量、提升质量，靠转型升级实现稳中有进。我们始终坚持改革为先，扩大开放。紧紧围绕重点领域和关键环节，纵深推进各项改革，努力破除体制机制障碍；大力实施开放招商，集聚各类发展要素，增强经济发展后劲。我们始终坚持统筹为策，协调推进。既全力稳定即期增长，又科学谋划长远发展；既大力改善基础条件，又持续提升发展环境；既注重经济发展，又注重生态建设和社会建设，发展的协调性和可持续性不断增强。我们始终坚持民生为本，利民惠民。把改善民生作为一切工作的出发点和落脚点，真心实意为群众办实事、做好事、解难事，让全市人民更多、更好地享受改革发展成果。我们始终坚持团结为重，积聚合力。各部门、各单位勇于担当、履职尽责，社会各界同心同德、团结协作，全市上下形成了干事创业、奋勇争先、共谋发展的浓厚氛围。这些宝贵的精神财富已经转化为推动发展的强大动力。

五年开拓奋进，五年成就辉煌。如今的洛阳，已经站在了一个新的历史起点上。这所有的一切，得益于省委、省政府和市委的坚强领导，得益于市人大、市政协的监督支持，得益于全市人民和社会各界的无私奉献。在此，我代表市人民政府，向辛勤工作在各条战线的全市人民，向人大代表、政协委员，向各民主党派、工商联、无党派人士和人民团体，向离退休老领导、老同志，向驻洛部队、武警官兵和公安干警，向所有关心支持洛阳发展的社会各界和海内外朋友，表示衷心的感谢并致以崇高的敬意！

在总结成绩的同时，我们更清醒地认识到，我市经济社会发展中还存在不少问题，主要是：产业结构不优，创新能力不强，战略新兴产业、现代服务业比重偏低，转型升级任务艰巨；城乡二元结构明显，农业基础依然薄弱，扶贫任务重，农民持续增收难，农民工市民化程度低，新型城镇化亟待破局；能源资源约束趋紧，环境保护特别是大气污染治理压力增大，传统发展模式难以为继；改革的力度和深度不够，制约发展的体制机制障碍亟待破解；民生欠账还较多，就业不充分及教育、医疗等资源不平衡的问题尚未得到很好解决；社会治理难度加大，安全稳定任务繁重；政府职能转变还不到位，机关作风、干部服务意识与群众期望尚有差距，在一些地方和单位“四风”问题还比较突出，发展环境需进一步优化。对此，我们将采取有力措施，认真加以解决。

二、今后五年主要任务

今后五年，是我市完成“十二五”规划目标、全面建成小康社会的关键时期，也是洛阳乘势而上、大有作为的重要战略机遇期。世界经济仍将延续缓慢复苏态势，我国经济长期向好的基本面没有改变，新一轮科技创新蓬勃兴起，产业转移加快推进，全面深化改革必将释放新的动力和活力。我市正处于工业化、城镇化快速发展阶段，中原经济区等三大国家战略全面实施，对我市扩大内需、加快产业升级具有重大的带动意义。洛阳改革开放30多年来的持续快速发展，近年来应对金融危机的艰苦实践，为我们加快发展奠定了基础、积累了经验。“开放招商、服务企业、优化环境”三大举措产生的积极效应逐步显现。特别是洛阳得天独厚的各种要素资源，更是打造洛阳经济“升级版”的独特优势。洛阳作为国家“一五”时期重点建设的老工业基地，工业基础雄厚，拥有一大批实力突出的骨干企业和高素质的产业工人，装备制造、石油及煤化工等传统产业正在创造新的竞争优势，电子信息、新材料等战略新兴产业正在快速成长，为我们推进新型工业化奠定了坚实基础。洛阳作为国家重要的科技研发基地，拥有一批国家重点实验室和技术拔尖人才，航空航天、装备制造、新材料、生物技术等领域研发水平全国领先，必将为洛阳经济转型升级提供新的动力。洛阳作为十三朝古都，文物古迹星罗棋布，文化底蕴十分丰厚，是我们建设“华夏文明传承创新核心区”的无可比拟的宝贵财富。洛阳作为中国“十大最具特色休闲城市”，山水生态禀赋优越，为我们“借水生财、点石成金”，打造休闲度假养老胜地提供了难得的资源。因此，我们一定要振奋精神，坚定信心，扩大已有优势、挖掘潜在优势，培育竞争优势，努力打造名副其实的中原经济区副中心城市！

今后一个时期，政府工作的总体要求是：全面贯彻落实党的十八大和十八届二中、三中全会精神，围绕建设名副其实的中原经济区副中心城市，坚持外引内改、外联内升、产业为基、产城融合，以提速增量、提质增效为目标，着力抓好开放改革、产业升级、盘活资源、改善民生，不断做大经济总量、提升发展质量和效益，促进经济持续健康发展、社会和谐稳定，努力建成中原经济区的重要增长极、文化示范区、最佳宜居地和开放创新城。

今后五年，市政府工作的总体目标任务是：

——建设产业优势突出的富强洛阳。改造提升装备制造、有色金属及深加工、石油及煤化工、硅光伏等传统优势产业，大力发展电子信息、新材料、新能源、生物医药等战略新兴产业，培育壮大现代服务业，积极发展特色高效农业，使洛阳的产业竞争能力、科技创新能力、区域竞争力和辐射力不断增强，开放度持续提高，保持在中原经济区城市的领先优势。到2018年，全市生产总值力争突破5000亿元，形成4个千亿级产业、20个左右的百亿级企业，打造洛阳经济“升级版”。

——建设独具特色的文化洛阳。坚持“文化立市”和“旅游兴市”战略，深入挖掘和利用文化资源优势，发展壮大文化旅游产业，以文化引领城市建设和发展，打造具有河洛特色、世界影响的国际文化旅游名城和著名旅游目的地城市，不断增强文化影响力、提升文化软实力，奠定“华夏文明传承创新核心区”的地位。到2018年，文化旅游产业增加值占生产总值的比重达到15%。

——建设便民高效的智慧洛阳。推动信息化与经济社会深度融合，大力发展和运用大数据、云计算、物联网等新一代信息技术，整合城市基础服务信息资源，实现在政务、商务以及交通、教育、医疗、城管、公安等领域的普及应用，建设2－3个在国内有重要影响力的智慧产业园，建成管理高效、服务便利、产业智能、绿色生态的智慧洛阳，提升社会治理能力。

——建设生态宜居的美丽洛阳。坚持以人为本、优化布局、注重生态、传承文化的原则，统筹推进城市建设、环境保护，实施林业生态圈建设，打造市区生态水系，加强农村环境综合整治，提升城乡基础设施和生态文明建设水平。到2018年，万元生产总值能耗下降15%以上，森林覆盖率和城市绿化覆盖率分别达到50%和40%，绿色经济初具规模，城市功能更加完善，城乡环境更加宜居，人与自然和谐相处，成为中原经济区最佳宜居地。

——建设平安和谐的幸福洛阳。加大民生投入力度，提高城乡公共服务均等化程度，促进教育、医疗、养老等领域保障能力提升。到2018年，城乡居民人均收入比2010年翻一番，人民生活基本达到小康水平，社会治理体制趋于完善，公共安全体系更加健全，法治化水平明显提高，群众安全感、幸福感进一步增强。

实现上述目标，必须准确把握形势，理清工作思路，突出工作重点：一是更加注重科学谋划，提升可持续发展空间。坚持以科学的战略规划引领经济社会发展，统筹考虑城市空间规划、功能分区、基础设施和产业布局。本届政府任期内要完成“十二五”规划的目标任务，编制实施“十三五”规划、新型城镇化规划，修订完善土地利用总体规划和综合交通体系规划、现代物流发展规划等重大专项规划，搞好规划衔接，使各项规划既有技术路线，又有项目支撑，确保政府决策更具科学性、执行更具可行性、发展更具长远性。二是更加注重转型发展，做到提速增量和提质增效并重。坚持以信息化带动工业化，以工业化促进信息化，大力引进资金、技术和优质资源，抓好老工业基地改造升级，提升装备制造业、振兴光伏产业、促进煤化石化产业融合发展、培育壮大新材料和电子信息产业，打造集群优势，构建现代产业体系，努力走出一条“科技含量高、经济效益好、资源消耗低、环境污染少、人力资源优势得到充分发挥”的新型工业化道路。以新型工业化引领和带动物流、文化旅游、金融等现代服务业和现代农业发展壮大，实现三次产业协同发展格局。三是更加注重城乡统筹，增强县域经济实力。围绕“全省经济社会发展的重要增长极，现代产业发展示范区、河洛文化旅游精品区、城乡统筹发展实验区、现代复合型新区和对外开放示范区”的目标定位，进一步完善洛阳新区的城市功能，培育支柱产业，努力打造最宜居、最具创新活力、最令人神往的城乡一体化示范区。以棚户区改造为带动，完善和提升洛北城区的城市功能，推动基础设施向周边延伸，适时进行区划调整，增强综合承载力和带动力。科学布局发展空间，推进县城提质扩容，增强产业集聚和人口吸纳能力。以人的城镇化为核心，破解户籍、住房、社会保障等体制机制障碍，促进农村人口向城镇转移，提高城乡一体化发展水平。四是更加注重改革创新，激发市场主体活力。以改革转变机制、破解难题，在一些重点领域、关键环节先行先试、取得突破。突出企业主体地位，坚持以思路创新带动机制创新、管理创新、产业创新和技术创新，提升核心竞争力。积极培育高科技产业，加快产学研军融合发展，引导社会资源和创新要素向企业流动，促进科技产业集聚发展。大力实施人才引进战略，培养引进一批科技领军人才、创新团队，打造人才高地，增强人才对经济发展的支撑能力。五是更加注重盘活资源，提升城市竞争力。坚持走特色城市发展之路，用战略思维和长远眼光经营城市，千方百计盘活资源，挖掘并发挥好洛阳现有的产业、科技、山水、生态、文化等优势，加强政策引导，优化资源配置，靠综合优势来聚人气、聚产业，全面提升城市竞争力和辐射力。六是更加注重改善民生，促进社会和谐稳定。坚持新增财力向民生倾斜，办好惠民实事，切实解决好就业、教育、医疗、住房、养老等群众最关心、最直接、最现实的热点问题。重视低收入群体生活，扎实推进扶贫开发，做好特困救助工作。维护好群众的合法权益，推进基本公共服务均等化,提升人民群众的幸福指数，让广大人民群众共享多享改革发展成果。

三、2014年工作安排

2014年是深入贯彻党的十八大、十八届三中全会精神的关键之年，也是新一届政府的开局之年。初步考虑2014年全市经济社会发展的主要预期目标是：地区生产总值增长9%左右；地方公共财政预算收入增长11%；固定资产投资增长22%；社会消费品零售总额增长14%；外贸进出口总额增长10%；城镇居民人均可支配收入和农民人均纯收入均增长9%以上；登记失业率控制在4.5%以内；居民消费价格涨幅控制在3.5%左右；人口自然增长率控制在6.5‰以内；节能减排完成省定目标。

（一）坚持优化结构、提升质量，努力推动工业经济持续稳定增长。注重以服务稳运行、以项目增后劲、以创新添动力、以载体强支撑，不断提高新型工业化水平。强化服务企业。完善服务企业工作机制，成立洛阳市企业服务办公室，以解决企业市场开拓、要素保障、政策落实等问题为重点，深入开展产销对接、银企对接、用工对接等服务企业活动。综合运用各类有效措施，加大对困难企业的帮扶力度。跟踪服务一批已投产未达产项目，着力解决突出问题，尽快形成新的经济增长点。强化项目带动。依靠项目调结构、促升级，推动传统优势产业向产业链和价值链高端延伸，重点抓好洛阳石化1800万吨炼油扩能改造、轴研科技伊滨产业园、中硅高科太阳能多晶硅切片、永金能化20万吨乙二醇等194个重点项目。加快培育壮大智能装备制造、电子信息、新材料、新能源、节能环保等战略新兴产业，积极推进沃德福工业机器人、厦门钨业高性能硬质合金等项目，确保中兴通讯智慧产业基地、力盛芯微光机电技术芯片等19个项目开工建设，洛阳单晶硅6英寸电路级硅抛光片等27个项目竣工投产。强化创新驱动。完善政府科技投入机制，强化企业科技创新主体地位，组建产业技术创新战略联盟，围绕产业链部署创新链，推动协同创新。谋划设立洛阳机器人与智能装备产业技术研究院，启动洛阳大学科技园二期建设，加快中意研究院、科技城等创新载体建设。组织高校、科研院所、产业集聚区和企业之间开展产学研合作对接，实施一批技术创新项目，促进科技成果转化。强化载体支撑。围绕主导产业链谋划建设项目，促进企业集群发展、产业集聚发展。年内产业集聚区建成区、发展区的主要基础设施要全部建成投用，公共服务平台基本完善，满足入驻企业在科技、就业、培训、信息等方面的服务需求。

（二）坚持扩大规模、增加总量，努力把服务业打造成经济发展的重要支撑。把壮大服务业作为调整产业结构、促进经济转型的关键举措，努力形成对经济发展带动力强、辐射面广的服务业发展格局。加快文化旅游业转型升级。科学编制文化旅游产业发展规划，完善公共服务体系。全面提升龙门石窟、白马寺佛教文化园区等重点旅游景区，启动中原影视文化产业园、白云山国家旅游度假区建设，加快推进老城历史文化街区、隋唐城“一区一轴”保护展示等重点文化旅游项目，打造核心文化旅游品牌。加快洛阳国家级文化和科技融合示范基地建设，谋划建设一批重点文化产业示范项目和示范园区，培育和发展文化创意、动漫游戏、数字出版等新兴业态。支持开发具有本地特色的文化旅游商品。优先发展现代物流和会展业。编制实施现代物流业发展规划，抓好中储物流、国龙物流、林安物流等一批综合性物流项目。办好第32届牡丹文化节，吸引更多的知名展会、赛会、年会落户我市，发展具有本地特色的品牌展会。大力发展信息服务业。抓紧申报国家电子商务示范城市、信息惠民示范城市，引进电子商务零售巨头，壮大中小网商队伍。大力培育软件开发、工业设计、服务外包等新兴业态，突出抓好惠普－洛阳国际软件人才及产业基地等18个专业园区建设。积极申报国家服务外包示范城市，努力打造中部地区具有较强竞争力的服务外包产业基地。做好全国互联网上网服务企业转型升级试点工作。提高金融服务业发展水平。整顿金融秩序，优化金融生态环境，引进金融机构2－3家，加快推进洛阳银行上市融资和多元化经营，鼓励金融机构在乡镇、社区设立分支机构和自助网点，推动企业上市和新三板挂牌融资。改造提升商贸流通业。利用电子商务改造传统商业模式，启动市区传统物流市场搬迁改造、提升工作。加快推进商务中心区和特色商业区建设，抓好长申国际购物广场、王府井购物广场等大型商贸项目，积极培育高端商务集群和大型商业综合体，推动供应链、运营、销售渠道、服务方式、盈利模式等方面综合创新。支持房地产业健康发展。认真落实房地产市场调控政策，引导企业开发质优价廉的商品房，促进商业地产、旅游地产和文化地产发展，满足居民和游客的多层次消费需求。积极发展健康服务业。充分挖掘特色资源优势，加快绿城健康养生休闲度假区等项目建设，培育中医保健、休闲养生、健身美容等新兴业态。积极创建国家养老服务业综合改革试点城市，鼓励民间资本投资养老产业。

（三）坚持强基固本、惠农富农，努力提升农业现代化水平。认真落实各项惠农政策，持续加大“三农”投入，努力走出一条生产技术先进、规模经营适度、市场竞争力强、生态环境可持续的新型农业现代化道路，让农业更强、农村更美、农民更富。培育壮大现代农业。进一步提高粮食综合生产能力，大力发展特色高效农业和畜牧业，不断拉长产业链条，加快建设一批农产品加工示范基地。发展壮大牡丹产业，提高产业综合效益。积极引进社会资本投入农业开发，鼓励扶持农民合作社、家庭农场、农业公司等新型农业经营主体，培育壮大产业化龙头企业。推进农产品电子商务、质量溯源等信息化试点和物流体系建设，建立农产品异地销售网络，打响洛阳特色农产品品牌。大力实施都市生态农业工程。积极发展观光农业、体验农业、休闲农业，在城乡一体化示范区和有条件的县城周边，规划建设一批基础好、功能全的都市生态农业示范园区。提升“菜篮子”工程质量。加快推进新农村建设。修订完善新农村建设规划，再启动建设一批美丽乡村示范点。持续实施乡村清洁工程，再完成30%的行政村环境集中整治达标任务。全面启动丘陵山区抗旱吃水工程，解决25万人口安全饮水问题。大力推进乡村通畅工程，新改建县乡公路200公里，硬化通组通户道路2000公里。全面深化扶贫攻坚。坚持“输血”与“造血”相结合，加快推进重点扶贫工程，支持贫困地区发展特色农业、特色旅游，构建专项扶贫、行业扶贫、社会扶贫“三位一体”的大扶贫格局。完成扶贫搬迁9091户，改造危房1万户，力争6.87万人实现脱贫。

（四）坚持城乡统筹、协调推进，努力加快新型城镇化进程。发挥规划引领作用，强化功能支撑，提高新型城镇化发展水平。完善洛河以北城区功能。继续推进城中村、棚户区改造，加快改善群众居住条件。开工建设新街跨洛河大桥、九都路东延长线、洛浦公园整体提升等重点工程，确保九都路改造等4项工程牡丹文化节前竣工、洛北城区东西环路等21项工程年底前竣工。抓紧改造城市地下管网，提高内涝防范能力。大力推进新区开发。洛阳新区要按照新的发展定位，科学编制规划，强化产城融合，率先实现城乡基础设施一体、生态共建、公共服务均等化的目标，努力打造生态宜居、创新发展的城乡一体化示范区。加快推进南环、西环、中原大道、高铁大道跨二广高速立交桥等项目建设，建成城乡一体化示范区骨干路网。抓好隋唐里坊区展示、万安山及龙门西山生态旅游综合开发等重点项目，大力推进商务中心区、龙门大道及高铁站周边等重点区域开发，完成智慧产业园区规划和样板项目建设。增强县城小城镇承载力。实施县城提质扩容工程，加大县城改造和新区建设力度，完善小城镇基础设施和配套服务功能，建设一批工业强镇、旅游名镇、现代农业特色镇。推动公共服务向乡镇、村庄延伸，形成带动周边农村的生产生活服务中心。强化区域发展支撑。完善综合交通体系规划，加快龙门高铁站枢纽、武西高速洛阳段、济洛高速等项目前期工作，抓紧实施311国道改造提升、客运南站等工程，确保洛栾高速洛龙站、连霍高速朝阳站牡丹文化节前建成投用，洛吉快速通道二期、连霍高速洛三段改造工程年底前竣工通车。抓紧完成机场改扩建工程，启动二期工程前期工作，完善检验检疫等口岸功能。积极推进城乡电网协同提升，确保220千伏西工变电站等一批电网工程尽早建成投用。加快热力管网建设，新增供热面积300万平方米。推进天然气“县县通”工程。提高城市精细化管理水平。做好智慧洛阳顶层设计，加快建设4G网络，统筹推进相关领域的信息化建设。实施数字化城市管理系统二期工程，逐步实现县（市、区）全覆盖。开展市容市貌提升和专项整治行动，所有主次干道全部实现机扫。继续实施公交增容，加快公交场站、人防过街通道及天桥建设，缓解城区交通拥堵。创新完善城镇化推进机制。拓宽投融资渠道，鼓励社会资本参与城乡基础设施建设。扩大保障范围，逐步将符合条件的进城务工人员及新就业的大中专毕业生纳入城镇住房保障体系。全面推行居住证管理制度，推动城镇公共服务常住人口全覆盖，让更多的进城务工人员真正融入城市。加快推进农村集体土地确权登记发证和“人地挂钩”试点工作，提高节约集约用地水平。

（五）坚持生态优先、绿色发展，努力建设山青水碧、环境良好的美丽家园。积极创建国家环保模范城市、生态园林城市和水生态文明试点城市，不断提高生态文明建设水平。强化环境污染治理。加强大气、水、重金属污染防治，减少雾霾天气。上半年全市30万千瓦以上燃煤机组脱硝工程全部建成。7月1日起所有火电机组全部执行新的污染物排放标准。年底前水泥、玻璃等非电燃煤行业全部实现达标排放，城市区燃煤锅炉和21台集中供热锅炉、各县（市）集中供热供气区域内的10蒸吨以下燃煤锅炉全部拆除或改用清洁能源，新的燃煤电厂项目一律不再审批；未通过环保检测的车辆全部实行重点区域限行。加大饮用水源地保护区环境整治和监管力度，启动新区第二污水处理厂和涧西污水处理厂扩容工程，确保已规划的县城第二污水处理厂、产业集聚区污水处理厂全部建成投用，所有重金属企业达标排放，城市区“四河七渠”排污口全部截流。全面提升节能水平。加快实施工业、建筑、交通、公共机构等节能降耗行动计划，抓好大唐首阳山电厂发电机组综合节能改造等重点项目，全面完成21个重点行业落后产能淘汰任务。积极创造条件，加快推进光伏电站建设。抓紧实施 “城市矿产”示范基地、企业节能技改资源综合利用等重点项目，开工建设城市垃圾综合处理园区，积极推进餐厨废弃物资源化利用和无害化处理试点城市建设，持续抓好秸秆禁烧和综合利用，加快资源再生型城市建设。大力推进生态建设。全面启动林业生态提升工程、中心城区林业生态圈建设和城市生态园林绿化提升专项行动，重点抓好城区周边绿化、组团绿化、通道绿化等项目，新增城市绿地330公顷，造林2.7万公顷，着力打造景观廊道、环城林带、城郊森林、生态园、湿地公园等多层次、立体化的绿色生态网络。加快实施洛河市区段东西延伸工程和涧河、瀍河、中州渠等治理工程，确保引黄入洛工程年内全线贯通。

（六）坚持深化改革、扩大开放，努力释放一切创造活力。深化国有企业改革，支持非公有制企业参与国企重组，积极发展混合所有制经济。启动21家驻洛央企“三供一业”分离移交和17户厂办大集体改革。制订非公有制企业进入特许领域的具体办法，支持民间资本进入基础设施、金融、民生等领域，鼓励非公有制企业建立现代企业制度、进一步做大做强。降低准入门槛，完善、落实信用评级、信贷风险补偿等扶持政策，积极培育中小企业公共服务平台，支持中小微型企业快速健康发展。创新政府融资平台管理，增强政府融资平台的资本运营、产业培育和风险管控能力，防范政府债务风险。积极参与中原经济区农村金融改革试验区建设。按照省政府部署，全面完成政府机构改革任务。推动教育、交通运输和行政执法体制改革。规范完善事业单位岗位管理，探索建立岗位动态调控机制。深化公立医院改革。扎实推进财税体制改革，扩大政府购买社会服务范围。理顺城市供热管理体制。

大力开展“开放招商提升年”活动。完善招商机制，全面落实政策，加强项目储备，加大项目跟踪服务力度，提高项目落地率。围绕重点产业，按照延链、补链、建链、强链集群发展的方式，强化以商招商、中介招商、大员招商、驻地招商，争取在战略新兴产业以及金融保险、现代物流、电子商务、服务外包、文化旅游、医疗、教育等领域引进一批战略合作项目。坚持吸引外资与利用内资并重、招商引资与招才引智并重，通过引进人才、技术、项目，带动产业发展，提高招商引资实效。努力扩大进出口贸易，优化出口结构，提高服务贸易发展水平。支持有条件的企业走出去发展。积极利用郑欧货运班列，主动融入国家丝绸之路经济带。

（七）坚持民生为本、和谐为基，努力让人民群众过上更好的生活。进一步加大财政投入，实施基本公共服务体系三年行动计划，扎实办好十大民生实事，提高城乡居民生活质量。推进教育现代化建设。抓好城市区幼儿园配套建设，重点发展农村学前教育，力争每个乡镇都建有一所普惠性幼儿园。提高义务教育均衡发展水平，加快普及高中阶段教育。实施美丽园丁教育基金项目，加强教师培训，全面提高教师和校长的教学研究水平。支持高等院校优化专业设置，整合职业教育资源，打造特色品牌，培育更多的高技能人才。推动实现更高质量的就业。完善城乡公共就业服务体系，加大创业孵化、技能培训力度，统筹促进高校毕业生、农村转移劳动力、退役军人、困难家庭等就业创业，新增城镇就业10万人。完善社会保障体系。继续推动社保扩面。完善被征地农民社保政策。提高城乡居民大病保险待遇水平。健全困难群众保障标准与物价上涨挂钩联动机制，逐步提高保障能力。完善临时救助机制，保障临时性、突发性困难家庭基本生活需要。开工建设各类保障性住房3.3万套，新增保障家庭6000户。完善养老服务体系，新建一批社区老人日间照料中心，改扩建一批农村敬老院。提高医疗卫生保障水平。继续推进优势医院倍增计划，加快实施河科大一附院、正骨医院、第二中医院等重点项目，提升公共卫生服务能力。完善医疗联合体和区域协作机制，健全基层医疗卫生服务体系，合理规划建设社区卫生服务站，实施居民就医“一卡通”工程，让群众看病更加便利。繁荣发展文体事业。积极创建国家公共文化服务体系示范区，加快市文化馆等公共文化设施规划建设，扎实开展文化惠民活动。加大文艺院团扶持力度，创作一批群众喜闻乐见的文艺精品。全面推进大遗址保护工程，继续做好丝绸之路和大运河申遗工作，筹建含嘉仓、回洛仓博物馆，启动汉魏故城博物馆、商城遗址公园项目，积极谋划二里头遗址公园项目。加快创建全省全民健身示范市，完善城乡体育设施，促进体育产业发展。持续加强平安洛阳建设。组织好村委会换届选举工作。稳步推进信访制度改革，创新信访体制机制，健全基层综合服务平台，依法解决群众合理诉求。加强网络社会管理，创造文明理性、规范有序的网络环境。强化社会信用体系建设，整顿和规范市场经济秩序。建立完善的食品药品监管体系，严格责任追究，确保群众食品药品安全。深入推进“安全洛阳创建”，加快尾矿库下游居民搬迁，加强对煤矿、危险化学品等重点领域的专项整治，杜绝重特大事故发生。创新社会治理方式，积极开展基层平安创建，构筑立体化的治安防控体系。认真做好新形势下的国防动员和民兵预备役工作，提高“双拥”共建水平。巩固提升创建成果，确保通过国家卫生城市、全国文明城市复审。支持工会、共青团、妇联等人民团体工作。继续做好民族宗教、外事侨务、港澳、对台、史志、档案、审计、地震等工作。

四、建设人民满意的政府

新的形势、新的任务，对政府工作能力、服务水平提出了新的要求。要扎实开展党的群众路线教育实践活动，认真解决“四风”问题，勤勉尽责、务实创新，不断开创政府工作新局面，为全市人民和外来投资者提供更加优质的服务、创造更加良好的环境！

坚持科学理政，打造学习型政府。大兴学习之风，建立健全经常化、普遍化的学习制度，积极营造公务员终身学习的良好环境。坚持学有所成、学以致用，努力把学习成果转化为破解难题、推进工作的实际能力。进一步解放思想，不断更新知识层次和思维方式，推动政府管理创新、制度创新、工作创新，确保政府工作更富创造性、更具科学性。

坚持为民勤政，打造服务型政府。全面准确履行政府职能，强化市场监管、公共服务、社会管理、环境保护职责，把政府工作着力点转到创造宜居宜业的发展环境上来，转到提供优质公共服务上来，转到维护社会公平正义上来。始终把群众满意作为第一标准，把更多精力放到服务发展上，把更多财力用到民生改善上，把更多的改革红利落到群众身上！

坚持放权简政，打造效能政府。深化行政审批制度改革，取消和下放行政审批事项，清理不必要的资质许可和资格认定，把该放的权力放到位，把该管的事情管住管好。推行权力清单制度，建立行政审批事项目录和行政职权目录。开展行政审批标准化试点，优化跨部门审批流程，推进许可事项联合审批、网上审批，做到咨询事项一次说清、办事程序一次讲清、能办事项一次办清，让审批流程更合理、项目落地更容易、群众办事更便捷！

坚持公开施政，打造阳光政府。制订投资领域负面清单，规范、公布所有涉企优惠政策并抓好落实，为企业投资经营提供便利。完善公共资源交易平台，逐步将水利、交通、医药、扶贫等项目引入平台交易。全面推进政务公开，深化预算决算、“三公”经费公开，及时主动公开环境污染、食品药品安全、安全生产等信息，扎实开展百姓问政活动，做到重大事项让群众知情、重大决策让群众参与、权力运作让百姓监督，让权力在阳光下运行。

坚持依法行政，打造法治政府。完善政府立法，提高立法质量和水平。落实领导干部学法用法制度，提高各级政府运用法律手段管理经济社会事务和妥善处理社会矛盾的能力。完善决策咨询、社会听证、重大事项评估等制度，健全权力运行制约和监督体系。有序推进相对集中行政处罚权试点工作，规范行政处罚裁量权和审批主体自由裁量权。自觉接受人大及其常委会法律监督、工作监督和政协的民主监督，主动接受社会公众、新闻媒体监督。认真办好人大代表议案、建议和政协委员提案。

坚持廉洁从政，打造清廉政府。加强党风廉政建设，认真落实中央八项规定和党政机关厉行节约反对浪费条例，规范办公用房、公务用车、公务接待管理，严格控制出国出境考察，禁止建设政府性楼堂馆所，财政供养人员、“三公”经费一律只减不增。全面推进政府重大项目、重要事项全程监察，加强政府采购资金、政府投资项目、国有土地使用权出让等管理与监督。敢于动真碰硬，铁腕问责，严厉查处违法违纪案件，以治庸提能力、以治懒增效率、以治散正风气！

各位代表！新的征程，承载新的梦想；重任在肩，更当百倍努力。让我们紧密团结在以习近平同志为总书记的党中央周围，在省委、省政府和市委的坚强领导下，开拓创新、团结奋进、务实重干，为建设名副其实的中原经济区副中心城市、推动中原崛起、河南振兴做出更大的贡献！
